# Insights into the Evolution of Cotton Diploids and Polyploids from Whole-Genome Re-sequencing

**DOI:** 10.1534/g3.113.007229

**Published:** 2013-10-01

**Authors:** Justin T. Page, Mark D. Huynh, Zach S. Liechty, Kara Grupp, David Stelly, Amanda M. Hulse, Hamid Ashrafi, Allen Van Deynze, Jonathan F. Wendel, Joshua A. Udall

**Affiliations:** * Biology Department, Brigham Young University, Provo, Utah 84602; † Plant and Wildlife Science Department, Brigham Young University, Provo, Utah 84602; ‡ Department of Ecology, Evolution, and Organismal Biology, Iowa State University, Ames, Iowa 50011; § Department of Soil and Crop Sciences, Texas AgriLife Research, College Station, Texas 77843; ** Seed Biotechnology Center, University of California-Davis, Davis, California 95616

**Keywords:** cotton fiber, comparative genomics, molecular evolution, allopolyploid

## Abstract

Understanding the composition, evolution, and function of the *Gossypium hirsutum* (cotton) genome is complicated by the joint presence of two genomes in its nucleus (A_T_ and D_T_ genomes). These two genomes were derived from progenitor A-genome and D-genome diploids involved in ancestral allopolyploidization. To better understand the allopolyploid genome, we re-sequenced the genomes of extant diploid relatives that contain the A_1_ (*Gossypium herbaceum*), A_2_ (*Gossypium arboreum*), or D_5_ (*Gossypium raimondii*) genomes. We conducted a comparative analysis using deep re-sequencing of multiple accessions of each diploid species and identified 24 million SNPs between the A-diploid and D-diploid genomes. These analyses facilitated the construction of a robust index of conserved SNPs between the A-genomes and D-genomes at all detected polymorphic loci. This index is widely applicable for read mapping efforts of other diploid and allopolyploid Gossypium accessions. Further analysis also revealed locations of putative duplications and deletions in the A-genome relative to the D-genome reference sequence. The approximately 25,400 deleted regions included more than 50% deletion of 978 genes, including many involved with starch synthesis. In the polyploid genome, we also detected 1,472 conversion events between homoeologous chromosomes, including events that overlapped 113 genes. Continued characterization of the Gossypium genomes will further enhance our ability to manipulate fiber and agronomic production of cotton.

We aligned re-sequencing reads to the existing D-genome sequence and discovered novel changes between the A-genomes and D-genomes in both diploid and polyploid plants. We identified single base differences throughout the genome between the diploid genomes and discovered that 978 genes of the D-genome reference sequence are consistently deleted in the A-genome. We discovered that approximately 900 Kbp of sequence in the polyploid genome have been converted from one genome to another in separate conversion events scattered across the genome. These discoveries help us better understand the dynamic nature of polyploid genomes and provide many avenues for further genomic research in cotton.

The genus *Gossypium* (cotton) includes approximately 45 diploid species that are divided into eight monophyletic groups, each designated by a single letter (“A” through “G” and “K,” hereafter referred to as genome groups) (Wendel *et al.* 2012). Ancient hybridization between A and D diploids resulted in a new allopolyploid (AD) lineage in the New World approximately 1–2 million years ago (Wendel 1989). Two of the descendant allopolyploid species—*Gossypium hirsutum* (AD_1_) and *Gossypium barbadense* (AD_2_)—as well as two African-Asian A diploids—*Gossypium herbaceum* (A_1_) and *Gossypium arboreum* (A_2_)—were each independently domesticated for their long, spinnable, epidermal seed trichomes. These four species collectively provide the world’s cotton fiber production, with more than 90% of this total being attributable to the cultivation of “upland cotton,” *G. hirsutum* (Wendel and Cronn 2003). Understanding the cotton genome is important for facilitating advances in crop variety development and utilization. In addition, insights into polyploid evolution in cotton may further our understanding of other polyploid crops.

Molecular studies and comparisons between diploid cotton species have revealed a genus with extraordinary genome dynamics. For example, there is a nearly three-fold variation in genome sizes among diploids (Wendel and Cronn 2003; Wendel *et al.* 2012), with the A-genome (1.7 Gbp) being nearly twice the size of the D-genome (0.9 Gbp), largely because of the proliferation of GORGE3 gypsy-like retrotransposons (Hawkins *et al.* 2006). Despite this size difference, comparative mapping studies have indicated that gene order and co-linearity have been largely conserved between the diploid A-genomes and D-genomes (Brubaker *et al.* 1999), with the corollary that most genome size diversity reflects variation in the rates of proliferation and deletion of repetitive elements (Hawkins *et al.* 2009; Grover and Wendel 2010). Molecular phylogenetic and dating studies indicate that the A-genomes and D-genomes diverged approximately 5–10 million years ago. The F-genome of *Gossypium longicalyx* diverged from the A-genome after the A–D divergence, making it a suitable outgroup for a comparison of the A-genome diploids.

The respective A and D diploid genomes are closely related to the two homoeologous genomes in allopolyploid cotton, A_T_ and D_T_ (“T” denotes tetraploid), because allopolyploidization is thought to have occurred during the mid-Pleistocene era, or 1–2 million years ago (Wendel 1989). Consequently, genome differences between diploids A_2_ and D_5_ serve as a fair approximation of the differences between A_T_ and D_T_ tetraploid genomes (Udall 2006; Flagel *et al.* 2012). Thus, the existence of models of the diploid progenitors of allopolyploid cotton provide powerful reference points for inference of homoeology (*e.g.*, of genes, transcripts, RNA-seq reads) in allopolyploid cotton. The recent publication of the genome sequence of the D-genome diploid (*Gossypium raimondii*; Paterson *et al.* 2012) allows for the development of new analytical and comparative approaches for the genomics of both diploid and polyploid cotton. For example, a tool was recently created to assign the sequence reads of allopolyploid cotton to their respective genome after mapping reads from A-diploids and AD-polyploids to the *G. raimondii* (D_5_) reference sequence (Page *et al.* 2013).

Combined with the rapid increase in available sequence data, these new genomic approaches may facilitate molecular and traditional improvement efforts of cotton. For example, analysis of reads from diploids mapped to a single genome reference provides a straightforward method to identify single nucleotide polymorphisms (SNPs) between and within genomes, because each alignment of reads has identical relative positions (Page *et al.* 2013). In considering the relationships among sequences from A-genome, D-genome, and AD-genome cotton species, it is useful to distinguish between two classes of SNPs. Briefly, homoeo-SNPs are fixed differences that distinguish (and hence diagnose) the A-genomes and D-genomes. Allele-SNPs, however, are traditional segregating polymorphisms within a single genome, between the two alleles of an individual accession (*i.e.*, heterozygosity) or between the corresponding homozygous alleles of different accessions. Allele-SNPs are those historically used by breeders to improve cotton cultivars in marker-assisted or genomic selection methods. Homoeo-SNPs add another layer to practical utility of allele-SNPs in that they provide a genomic feature to distinguish between duplicate gene copies. Homoeo-SNPs are also useful in an evolutionary context because their analysis offers insights into the molecular evolutionary properties of allopolyploid cotton and, more generally, allopolyploid genomes.

To better understand both diploid and allopolyploid cotton genomes, we performed deep whole-genome re-sequencing of several diploid accessions of both A-genome and D-genome diploids. Our first objective was to determine all of the homoeo-SNPs between the A-genomes and D-genomes. Using reads from these diploids and publicly available reads from diploid and allopolyploid cottons, we compiled a database of SNPs between the various genomes studied. Our second objective was to describe genome evolution between the genomes that could be characterized by read coverage. We examined loci that are either duplicated or deleted in the A-genome species, based on coverage of A-genome reads mapped to the D_5_-genome reference. Where those duplications or deletions overlap with genes, they may provide insight into the evolutionary basis for the phenotypic differences among diploids, including the production of spinnable fiber in A-genome diploid species. Our third objective was to document the extent of genome interaction based on sequence data in the polyploid (*i.e.*, conversion events). A robust description of conversion events throughout the cotton genomes will serve as a bioinformatic aide to future genomic analyses of allopolyploid cotton.

## Materials and Methods

### Plant material

Plant material was grown and harvested from greenhouses at Brigham Young University (D_5_-2, D_5_-31, A_1_-155, A_2_-34, A_2_-1011), Iowa State University (D_5_-4, D_5_-53, A_2_-4, A_1_-73), and Texas A&M University (A_2_-255). DNA was extracted from four accessions of *G. raimondii* (D_5_-2, D_5_-4, D_5_-31, D_5_-53), two accessions of *G. herbaceum* (A_1_-73, A_1_-155), and four accessions of *G. arboreum* (A_2_-4, A_2_-34, A_2_-255, A_2_-1011) using a Qiagen DNeasy plant kit.

### Acquisition of DNA sequence

After shearing DNA with a Covaris instrument at the Huntsman Cancer Institute (Salt Lake City, UT), DNA libraries were prepared with the Illumina TruSeq V3 kit and sequenced by Beijing Genome Institute (BGI, Sacramento, CA), producing 100-bp paired-end reads. We assumed that the Illumina library construction process would perform equally well on high-quality DNA of the A-genomes and D-genomes. Approximately 40-times the genomic coverage was obtained for each library (Table 1). Reads from the diploids have been deposited in the NCBI Sequence Read Archive (SRA) under the following entries: PRJNA202235, PRJNA202236, and PRJNA202239 for *G. arboreum*, *G. herbaceum*, and *G. raimondii*, respectively. Additional genomic sequence reads for *G. longicalyx* (F_1_-1; SRR617255), *G. herbaceum* (A_1_-97; SRR617256, SRR617284, SRR617704), and *G. hirsutum* cv. Maxxa (SRR617482) were obtained from the Sequence Read Archive. All reads were trimmed for quality with Sickle using a minimum phred quality threshold of 20 (https://github.com/najoshi/sickle).

**Table 1:** Transitions and transversions in the homoeo-SNP index

	A	G	C	T
A	—	2,495,527	626,075	1,003,583
G	2,547,739	—	353,034	647,148
C	644,840	352,239	—	2,544,261
T	1,003,739	628,050	2,492,619	—

Rows = A allele. Columns = D allele. There was an overall transition/transversion ratio of ∼1.92 and GC fractions of 45.4% (A genome) and 45.1% (D genome).

### Homoeo-SNP index

An index of homoeo-SNPs between the A-genomes and D-genomes was produced by comparing sequence data from nine *Gossypium* diploids (A_1_-97, A_1_-155, A_2_-34, A_2_-255, A_2_-1011 *vs.* D_5_-2, D_5_-4, D_5_-31, D_5_-53). First, all reads were mapped with GSNAP (Wu and Nacu 2010) using the options “-n1–Q” (requiring unique best mapping for each read) to the 13 chromosomes of the D_5_ reference sequence (Paterson *et al.* 2012). Second, alignment files were processed with SAMtools to produce sorted BAM files (Li *et al.* 2009). Third, we used InterSnp, a custom code built on the BAMtools API (https://github.com/pezmaster31/bamtools) and available as part of the BamBam package (http://udall-lab.byu.edu/Research/Software/BamBam.aspx) to call SNPs with at least 10-times coverage and a minimum minor allele frequency of 40%. Because the alignments from diploids used the same reference genome, homologous loci in the A-genomes and D-genomes were readily compared to identify SNPs between genomes (homoeo-SNPs). Homoeo-SNPs were called at a locus position of the D-genome reference when all diploid genomes with coverage at that particular locus were homozygous, all A-genome diploids had the same base, and all D-genome diploids had the same base, different from the A-genome diploids. Finally, loci with identified homoeo-SNPs were tabulated into a text file that was converted into a homoeo-SNP index for use by GSNAP and PolyCat.

### SNP identification and diversity analysis

Using the homoeo-SNP index, we again mapped the sequence reads from the nine diploids, this time using the SNP-tolerant mapping (“-v” option) of GSNAP. We also mapped reads for three additional diploids (F_1_-1, A_1_-73, A_2_-4) and one allopolyploid (*G. hirsutum cv. Maxxa*). GSNAP and SAMtools were otherwise used as noted. Reads from the tetraploid Maxxa were assigned to the A_T_-genomes and D_T_-genomes using PolyCat (Page *et al.* 2013). All SNPs (homoeo-SNPs and allele-SNPs) were called by InterSnp between the 13 resulting BAM files, one for each A or D diploid, one for the A_T_-genome of Maxxa, and one for the D_T_-genome of Maxxa. The number of heterozygous loci in each individual was summarized after filtering loci within conserved duplications. We constructed a neighbor-joining tree for the various diploid accessions, as well as the A_T_-genomes and D_T_-genomes, using the PHYLIP (Felsenstein 1989) program “neighbor” and default settings. The distance matrix consisted of the percentage of aligned sites that differed in pairwise comparisons.

We used homoeo-SNPs between the diploids to generate a “pseudo-A” genome, with the A alleles substituted into the D_5_ reference. We did the same with the Maxxa homoeo-SNPs to make “pseudo-A_T_” and “pseudo-D_T_” genomes. Although these pseudo-genomes did not have indels or structural variations that are present in the actual A, A_T_, and D_T_ genomes, the majority of gene sequences were conserved (Flagel *et al.* 2012; Paterson *et al.* 2012). Thus, these pseudo-genomes served to characterize the location of allele-SNPs within genes and other conserved noncoding sequences, and having each genome on the same "scale" greatly simplifies genome comparisons.

### Duplications and deletions

We detected putative duplications (relative to the D_5_ reference genome) in the other diploids using MACS (with default settings), a commonly used tool for ChIP-seq analysis (Zhang *et al.* 2008). It empirically models peaks in coverage of ChIP-seq reads using a dynamic Poisson distribution, thereby estimating the location of a DNA binding molecule. Here, we used MACS to call coverage peaks within WGS reads from the A-genome and F-genome diploids after alignment to the D-genome reference. Assuming the libraries from both A-genomes and D- genomes would be equally biased, reads from D_5_-53 served as a control sample, estimating the expected coverage pattern. Peaks in A-genome coverage relative to D_5_-53 represent putative duplicated sites that were sampled at a higher frequency during sequencing. To filter out false-positives, we also called coverage peaks in D_5_-2, D_5_-4, and D_5_-31, relative to D_5_-53. We used bedtools (Quinlan and Hall 2010) to compare peaks among and between the datasets and to identify their position relative to gene annotations in the D_5_ version 2.1 (Paterson *et al.* 2012).

Similarly, putative deletions in the test diploids were called if the reference sequence D_5_-53 had 20-times or higher coverage at both ends, as well as at an additional point at least 200 bp from either end of a region of at least 1000 bp, and if the test diploid had near-zero coverage (<3-times) at every point in that block. This detection was performed by Gapfall, part of the BamBam package (http://udall-lab.byu.edu). Blast2Go was used for an enrichment analysis (using the Fisher exact test) on genes duplicated or deleted in the A diploids (Conesa *et al.* 2005). Default B2G parameters were used.

### Polyploid conversion events

We used two methods to identify possible nonreciprocal homoeologous or “gene conversion” events between the A_T_-genomes and D_T_-genomes of *G. hirsutum* cv. Maxxa. We first identified individual converted loci based on homoeo-SNPs, where reads from the A_T_-genome carried the D-genome nucleotide, or vice versa. The second method used duplications and deletions to identify regions of conversion, but for deletion detection using 15-times the minimum coverage for the duplicated genome and less than four-times the coverage for the "deleted" genome. If a region spanning at least 1 Kbp was “duplicated” in the A_T_-genome relative to the A diploids and “deleted” in the D_T_-genome relative to the D diploids, then an A_T_-biased conversion event was inferred. Similarly, an A_T_-genome deletion and D_T_-genome duplication suggested a D_T_-biased conversion. These analyses of the polyploid genome were limited to regions that were present in both diploid genomes (A and D) because homoeo-SNPs could only be predicted in such regions.

## Results

### Intergenomic SNPs

**Figure 1:**
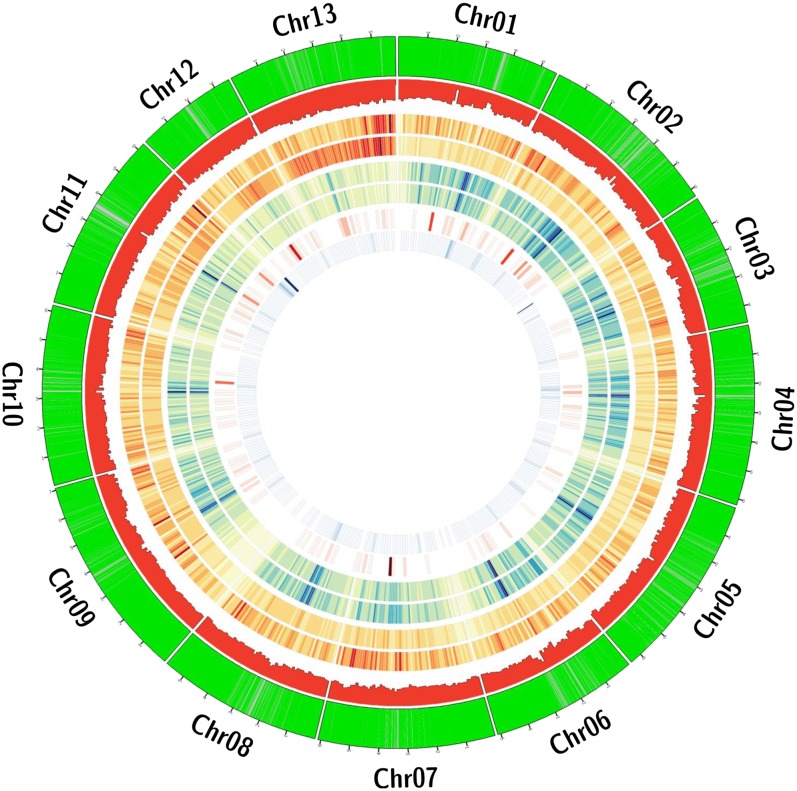
Plot of genes, homoeo-SNPs, duplications, deletions, and conversion events in the A-genomes, relative to the D_5_ reference sequence, produced by Circos (Krzywinski *et al.* 2009). Considering the concentric circles from the outside inward, the outermost (and first) green circle indicates the location of annotated genes. The next circle (red) is a histogram of the number of homoeo-SNPs in a 1-Mbp window throughout the genome. The next two red (high-frequency) to yellow (low-frequency) circles are heat maps showing the location of duplications in the A_1_ and A_2_ genomes as compared to the D_5_ genome (A_2_ interior). The next two blue (high-frequency) to yellow (low-frequency) circles are heat maps showing the location of deletions in the A_1_ and A_2_ genomes as compared to the D_5_ genome (A_2_ interior). The final two circles show conversion events in the tetraploid *G. hirsutum cv. Maxxa*. The first circle shows conversion of loci to the A nucleotide on a red-to-yellow scale, whereas the innermost (and last) circle shows conversion of loci to the D nucleotide on a blue-to-yellow scale.

Similar to previous work on the detection and frequency of SNPs in genic regions (Page *et al.* 2013), we produced a robust index of 23,859,893 homoeo-SNPs between the genomes of diploid A-genome and D-genome cotton. These SNPs covered the genome of the D-genome reference sequence at a density of one SNP per 32.3 bases (Figure 1). This total number of SNPs is a dramatic increase from the number previously reported with the D-genome sequence (Paterson *et al.* 2012) and in genic sequences (Page *et al.* 2013). The index had a transition/transversion ratio of 1.92 (Table 1), similar to the Maize HapMap2 (Chia *et al.* 2012). This genome-wide SNP analysis confirmed our speculation that the previous ratio was downwardly biased in our gene-focused index. Across polymorphic nucleotide positions, there was not a significant difference between the GC biases of the A-genomes and D-genomes (45.4% and 45.1%, respectively). However, these values were higher than the genome-wide GC content, suggesting an increased likelihood for SNPs at G or C nucleotides, possibly because of the high frequency of C→T mutations caused by de-amination of cytosines.

The genome-wide SNP index (SNP index 2.0) was based on comparisons of deep sequence coverage between multiple diploid A-genomes and D-genomes, so we anticipated it would be more robust and widely applicable for read mapping efforts of other diploids and allopolyploids than the previous index. The improved index increased mapping efficiency of A-genome reads to the D-genome reference sequence. With the SNP-tolerant mapping of GSNAP, more than 77% of A-genome reads mapped, reflecting a mapping improvement of approximately 15% compared to mapping without the SNP-index (Table 2, Supporting Information, Figure S1). D-genome mapping was unaffected (∼95%). The error rate of categorization of WGS reads was less than 2%, as estimated by categorizing the diploid reads and looking for incorrectly categorized reads. Although this error rate is slightly higher than that estimated for the original PolyCat index (Page *et al.* 2013), it is still an acceptable rate considering the increased fraction of reads that overlap a SNP between genomes (∼70%, up from <50%), and considering WGS reads mapping to less conserved intergenic regions.

**Table 2:** Summary of nine diploid WGS re-sequencing libraries that were re-sequenced in this study and additional libraries (A1_97, F1_1, and Maxxa) obtained from the SRA

Accession	PI	Raw Pairs	Trimmed Reads	Raw Mapping %	Mapped % Using SNP Index 2.0
A1_155	630024	385,657,228	761,269,884	65.3	78.0
A1_73	485587	202,723,343	238,035,929	53.1	85.6
A1_97	529670	328,713,056	652,350,335	65.0	77.8
A2_1011	629339	412,420,252	816,274,495	58.3	73.8
A2_255	615756	300,406,057	595,289,591	61.1	75.5
A2_34	183160	367,844,399	729,370,248	62.3	76.5
A2_44	185788	78,180,657	153,728,823	63.3	76.9
A2_4	529707	343,470,023	686,940,046	48.9	82.4
D5_2	530899	152,913,856	304,706,886	95.6	95.3
D5_31	530928	217,334,954	428,323,703	95.8	95.5
D5_4	530901	310,387,080	616,432,521	95.1	94.8
D5_53	530950	188,469,224	375,193,268	96.2	96.0
F1_1	530986	534,258,839	1,055,751,863	71.1	79.1
Maxxa Acala	540885	463,761,132	919,898,042	72.5	79.8

Within the diploid index, genes had a median intergenomic SNP per base rate of 2.2% (range, 0–16.1%). Notably, there were 593 genes that had no unambiguous homoeo-SNPs between diploid A-genomes and D-genomes (Figure S2). Of these, 215 genes had one or more allele-SNPs (*i.e.*, one diploid genome had two nucleotides, one matching the second diploid genome and the other nucleotide being novel). The remaining 378 genes were completely conserved across all accessions with no SNP differences. A Blast2Go enrichment analysis of these genes identified the following three enriched GO terms: NADH dehydrogenase (ubiquinone) activity (GO:0008137); NADH dehydrogenase (quinone) activity (GO:0050136); and NADH dehydrogenase activity (GO:0003954). Most of these genes were shorter than the average gene within the D-genome annotations (95 to 8113 bp with mean 810 ± 786 SD for the 378 genes *vs.* 89 to 51,174 bp with mean 3249 ± 2806 SD for all 37,223 genes; Figure S2) (Paterson *et al.* 2012).

In the polyploid, improved categorization of reads into its two separate genomes was enabled by the genomic SNP index. Using the SNPs from the diploids and the D-genome reference, PolyCat assigned more than 70% of mapped polyploid reads to the A_T_-genomes or D_T_-genomes (Figure S3). For the tetraploid Maxxa, a greater percentage of reads were assigned to the A_T_-genome than to the D_T_-genome, despite the fact that categorization only occurred in regions shared by the two genomes. This later criteria preempted the larger A-genome from an A_T_ categorization bias. The unexpectedly higher categorization rate of A_T_ reads may be partially explained by the fact that A_1_ and A_2_ diploids are a two-fold better approximation of the A_T_-genome than D_5_ is of the D_T_-genome. For example, nucleotide diversity appears to play a role in mapping efficiency among the diploid A-genome species. The most divergent line (A_1_-73) had the lowest mapping percentage of any of the of the A-genome diploid accessions. Because sequence divergence is less between the A-genome diploid and polyploid than the D-genome and polyploid, read categorization based on SNPs between the diploids would be more effective for the A_T_-genome, resulting in the observed bias. To a much lesser degree, the A_T_ categorization bias may also be partially attributed to duplicated loci in the A_T_-genome mapping to a single locus in the D_5_ reference, although these artifacts were largely avoided by the detection of duplications.

Because of their recent common ancestry, many of the identified differences between the A-genome and D-genome diploids were retained between the A_T_-genomes and D_T_-genomes as homoeo-SNPs. A total of 20,828,020 homoeo-SNPs were identified between the A_T_-genomes and D_T_-genomes of the allopolyploid cultivar Maxxa. The difference between the number of approximately 20 million SNPs in the polyploid and the “retained ancestral” homoeo-SNPs (∼16 million; 75.8%) were autapomorphic SNPs that were derived after the divergence of the A_T_-genome and D_T_-genome from the A-genome and D-genome common ancestor, respectively. This portion of the homoeo-SNPs (5,046,151; 24.2%) was only identified between the genomes in the allopolyploid and not in the comparison of the diploid genome sequences. These unique, homoeo-SNPs were found throughout the genome in 34,810 of the 37,223 annotated genes. We anticipate that additional polyploid autapomorphic SNPs will be identified as more polyploid genomes are re-sequenced.

For all of the annotated genes in the D-genome reference, an alignment of A, A_T_, D_T_, and D-genomes was created, from which the amount of molecular evolution between the A-genomes and D-genomes of cotton was calculated (Table 3). The results of this effort concurred with our previously published work based on aligned EST contigs (Flagel *et al.* 2012), although SEs were much smaller because of the much larger dataset. We found slightly less divergence (dN and dS) between the polyploid genomes (n = 28,317) than between the diploid genomes (n = 30,874), although the difference is not significant. The different totals between the diploids and polyploids suggested that more than 2500 genes in the polyploid did not have sufficient polymorphism for an appropriate estimation of molecular evolution. We further investigated the alignments of these genes to ascertain whether their close sequence similarity was the result of gene conversion between homoeologous genomes. Of the genes without dN/dS estimates, 759 were found to only have sufficient polymorphisms between the tetraploid genomes (and not between the diploid genomes) and 3316 were found to have sufficient polymorphisms between the diploids (but not between the tetraploid genomes). This cumulative large difference between ploidy levels further suggested that gene conversions may play a role in reducing genetic diversity between genomes. However, only 106 and 42 genes were detected to overlap "conversion regions" in the diploids and tetraploid genome.

**Table 3:** Amount of molecular evolution between the A and D genomes of cotton

		dN	dS	dN/dS
A *vs.* D	Mean	0.0094	0.0276	0.3726
n = 28,462	Median	0.0068	0.0256	0.2768
	SD	0.0106	0.0225	0.4236
A_T_ *vs.* D_T_	Mean	0.0092	0.0266	0.3772
n = 26,156	Median	0.0066	0.0237	0.2843
	SD	0.0104	0.0228	0.4156

**Figure 2:**
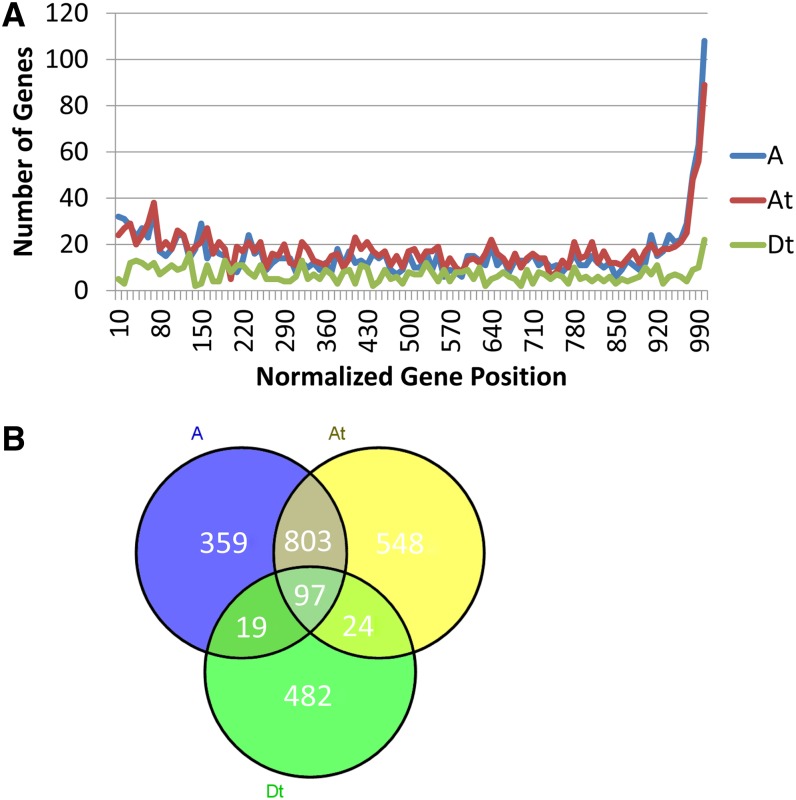
Premature stop codons were found in each *Gossypium* genome. (A) Premature stop codons (compared to the annotations of the D-reference genome) were found in the A, A_T_, and D_T_ genomes. (B) Common genes with premature stop codons in the first 90% of the gene.

Of the 2,817,991 SNPs between diploids that fell within genes, 486,514 were inferred to be in exonic positions, including 248,599 that caused amino acid changes (*i.e.*, nonsynonymous) compared to the reference sequence. Of these, there were 1651 genes with SNPs that resulted in premature stop codons in the pseudo-translation of A-genome transcripts, 1802 genes with premature stop codons in A_T_, and 709 genes with premature stop codons in D_T_ (Figure 2)_._ These genes were not excluded in estimates of molecular evolution. None of these gene sets had any enriched GO terms. The low level of D_T_ premature stops may simply reflect an ascertainment bias of the annotated reference genome that was based on a diploid D genome. Many of the putative stop codons were found near the annotated end of the gene, suggesting that they might have only a minimal impact on protein function. Alternatively, their inference may reflect bioinformatic artifacts, such as imperfect gene annotation of the D-genome, or alternative stops that independently evolved in the A-genomes. Most of these alternative stops codons were within 10% of the 3′ end of the gene. If one ignores the premature stop codons within the last 10% of the annotated genes, then 803 premature stops were shared between the diploid A-genomes and the A_T_-genome (Figure 2). This result was marginally less than previously reported (Paterson *et al.* 2012), because we had the added power of multiple A-genome re-sequencing efforts.

Other SNPs disrupted a start or stop codon. We identified 806 genes with disrupted start codons (*i.e.*, resulting in an amino acid distinct from that in the D_5_ reference) in the A-genome, 703 in A_T_, and 684 in D_T_. These genes could have a longer or shorter coding sequence than as originally annotated. No GO terms were enriched in these gene sets. We also identified 831 genes with altered stop codons in the A-genome, 693 in A_T_, and 437 in D_T_, resulting in longer peptide sequences. Several GO terms (∼20 in each genome) were enriched within these genes, and almost all were associated with photosynthesis (Table S1). There were also 406 genes without a stop codon within the D_5_ gene annotation, with the same photosynthesis GO terms being enriched.

### Diversity and heterozygosity

In addition to creating an index of nucleotide differences between the diploid A-genomes and D-genomes, we detected unique nucleotide variation within and between individual accessions. Within a genome type (*i.e.*, A or D), these types of SNPs are called allele-SNPs. The allelic genotype of each diploid and both genomes of the allopolyploid Maxxa were determined at all polymorphic loci. A pairwise comparison between accessions found that the D_5_ diploids had extremely low nucleotide diversity (<1 million SNPs) between any two accessions, whereas a similar pairwise comparison between the A_1_-genomes and A_2_-genomes found that accessions were more diverse (4–5 million SNPs within A_1_ or A_2_; 6–8 million SNPs between A_1_ and A_2_; Table S2). There were approximately twice as many SNPs between the A_1_-genomes and A_2_-genomes as within either of the two species. These results are not unexpected given the exceptionally low diversity found in a survey of allozyme diversity in *G. raimondii* (J. F. Wendel, unpublished data) and the appreciable levels of diversity in the chosen accessions of *G. arboreum* and *G. herbaceum* (Wendel *et al.* 1989).

In addition to having more fixed allele-SNPs between accessions, the A-genome diploids were more heterozygous than the D-genome diploids (∼13% and <1%, respectively; Table 4). In the A-genome diploids, heterozygous loci were approximately twice as frequent outside than inside of genes. This was not surprising, given the expectation of more intense purifying selection on coding sequences. Of course, these estimates of heterozygosity excluded loci that were duplicated in the A-genome. Interestingly, heterozygous loci in the D-genome diploids were equally common in genic and nongenic regions. This genomic difference likely reflects both the exceptionally low genetic diversity within the D-genome and a high level of generalized inbreeding. In this respect, we note that *G. raimondii* has a narrow natural range and presently exists as only scattered populations with very low effective population sizes.

**Table 4:** Number of heterozygous loci in each accession, along with the percentage of total observable loci that were heterozygous

	Whole Genome		Genic Loci Only		Nongenic Loci Only	
Accession	n	%	n	%	n	%
F1_1	9,968,998	17.2	332,247	6.1	9,636,751	18.4
A1_73	2,963,374	7.1	126,260	2.6	2,837,114	7.7
A1_97	6,504,768	12.4	265,607	5.0	6,239,161	13.2
A1_155	7,549,531	13.9	322,095	6.0	7,227,436	14.8
A2_4	7,061,224	13.2	283,151	5.3	6,778,073	14.1
A2_34	6,826,660	12.9	270,384	5.1	6,556,276	13.7
A2_255	5,898,387	11.6	236,113	4.5	5,662,274	12.4
A2_1011	6,878,801	13.1	252,230	4.9	6,626,571	14.1
D5_2	193,418	0.3	20,536	0.4	172,882	0.3
D5_4	257,399	0.4	25,370	0.5	232,029	0.4
D5_31	178,290	0.3	20,723	0.4	157,567	0.3
D5_53	181,224	0.3	20,665	0.4	160,559	0.3
Maxxa.A	4,465,088	9.2	198,477	3.9	4,266,611	9.8
Maxxa.D	686,674	1.3	47,861	1.0	638,813	1.4

**Figure 3:**
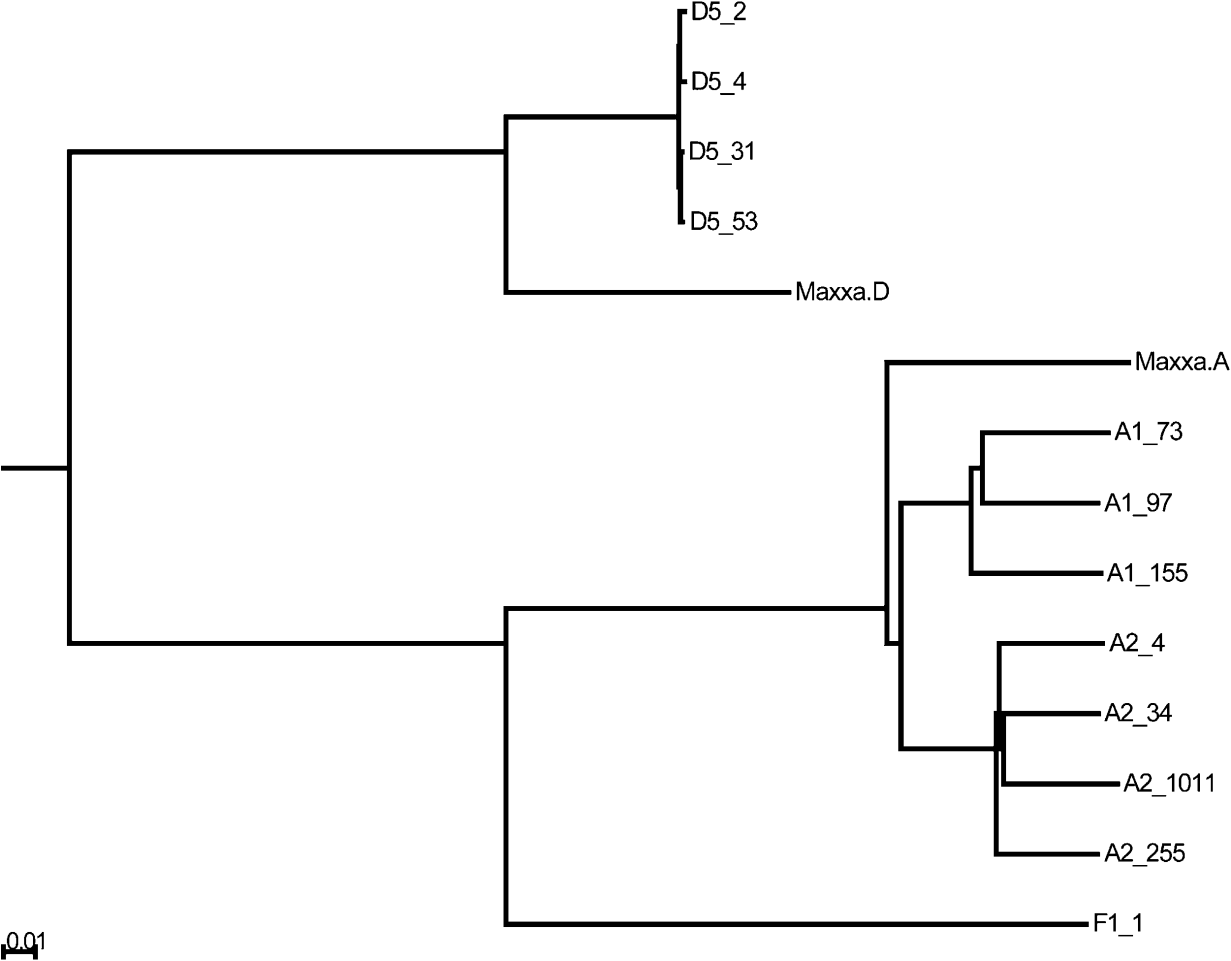
Neighbor-joining tree built by PHYLIP, based on SNPs between genomes. Units (as measured by the indicated scale) are percentage of represented polymorphic sites that differed between two individuals. Image rendered by Archaeopteryx (Han and Zmasek 2009).

A neighbor-joining tree was constructed and rooted based on the known relationship between the A/F-genome clade and the D-genome clade (Figure 3). Many fixed allele-SNPs (*i.e.*, not heterozygous within a line) could be attributed to mutations occurring along specific branches of the phylogeny (Table 5). The tree correctly reconstructed the accepted relationships of the diploids and their relationships to the two genomes of allopolyploid cotton (Senchina *et al.* 2003; Grover *et al.* 2012; Wendel *et al.* 2012). Specifically, and unsurprisingly, A_T_ and D_T_ were phylogenetically sister to the common ancestor of the [A_1_ + A_2_] clade and the D_5_ clade, respectively. Our results agreed with previous reports that the A_1_ or A_2_ diploids were approximately twice as good of an approximation of the A_T_-genome as the D_5_ diploids were of the D_T_-genome (Wendel and Cronn 2003; Senchina *et al.* 2003). The distance from D_T_ to D_5_ was 0.14, and the distance from A_T_ to the common ancestor of A_1_ and A_2_ was 0.08. However, the distances from A_T_ to A_1_ and A_2_ were 0.10 and 0.11, respectively. Moreover, the distance from A_T_ to any individual A-genome diploid was 0.14, similar to the distance between D_T_ and any D-genome diploid. Although the group of A-genome diploids provided an approximation of A_T_ that was two-times better than a group of D_5_ diploids did of D_T_, any individual A or D diploid appeared to be equally similar to its A_T_ or D_T_ counterpart. The exceptionally low diversity among D_5_ diploids explained the fact that a group of D_5_-genome diploids was not significantly better for approximation of the D_T_-genome than was a single D_5_. However, multiple accessions of A diploids (A_1_ and/or A_2_) did provide a substantial (nearly two-times better) improvement in construction of the A_T_-genome pseudo-sequence.

**Table 5:** SNPs attributable to specific areas of the phylogeny

Genome	SNPs	Deletions
All A	5,544,440	25,408
A_1_	1,024,299	3809
A_2_	1,152,825	2941
A_T_	1,472,900	5247
All D	14,601,331	0
D_T_	3,563,979	4518

As shown in Figure 3. Because of possible conversion events, it is not possible to determine how many SNPs were shared by all A or D diploids.

### Duplications and deletions

Duplications were detected in A-genome diploids as coverage peaked across the D-genome reference sequence (Figure 1). Because these duplications were detected relative to the D-genome diploids, they represent events that occurred after the split of these two clades 5–10 million years ago (Wendel and Cronn 2003; Wendel *et al.* 2012). Thus, peaks shared by all A-genome accessions represent pieces of the A-genome that are duplicated relative to the D-genome. These coverage peaks represent a mix of tandem and dispersed duplications, because the methodology used makes no distinction regarding genomic location of duplicated segments. There were 30,709 regions duplicated in all A-genome diploids but no duplication in D-genome diploids. These duplicated blocks overlapped 1007 genes, with a minimum overlap of 50% of the gene length. Only one GO term was enriched among these genes: structural constituent of ribosome (GO: 0003735).

In contrast to duplications, putatively deleted regions of the A-genome were detected with a higher degree of certainty because their diagnosis is based on lack of coverage rather than a quantitative difference in coverage (Figure 1). Some regions of the D-genome reference genome did not have any A-genome reads mapped to them, despite 40-times the WGS coverage based on the number of produced A-genome reads and correctly mapping D-genome reads to the same region. Each accession had a unique set of deleted regions, including genes (Figure S4). There were 25,408 regions deleted in all A-diploid genomes. The genomic regions included 978 annotated D-genome reference genes where the deleted region minimally overlapped 50% of the gene length. Among the genes within deleted regions, 118 GO terms were enriched compared to the population of GO terms within the annotated gene set (Paterson *et al.* 2012) (Table S3). Most of these deletion terms were associated with starch synthesis, tRNAs, or DNA repair mechanisms. Three hundred seventy-eight genes were completely deleted in the A-genome diploids relative to the D-genome diploids, meaning that the genic region was spanned by a single deletion block.

### Polyploid conversion events

We used two different methods to detect conversion events in the allopolyploid genome of Acala Maxxa (*G. hirsutum*). The first method identified historical nonreciprocal homoeologous recombination events (NRHR) at individual loci based on homoeo-SNPs within a polyploid genome. Earlier analyses in cotton used this method to detect conversion events (or NRHR events) based on comparative analysis of assembled EST sequences from diploid and allopolyploid cotton (Salmon *et al.* 2010; Flagel *et al.* 2012). The second method identified converted regions based on coverage patterns in the A_T_-genomes and D_T_-genomes relative to their respective diploid relatives. Here, we consider the NRHR events as “conversion” events regardless of whether they occur in coding or in noncoding sequences (*sensu amplo* of gene conversion).

Based on homoeo-SNPs (first method), 1,748,889 conserved SNPs in 29,576 genes in the diploid genomes suggested an A_T_-biased allele conversion (a D_T_ nucleotide converted to the A_T_ nucleotide). In contrast, a total of 361,795 SNPs in 12,346 genes suggested a D_T_-biased conversion. These data suggest a nearly five-fold bias based on homoeo-SNPs and a two-and-one-half–fold bias based on genes in favor of A_T_-biased conversion, in stark contrast to the two-fold bias in favor of D_T_-biased conversion reported previously (Paterson *et al.* 2012).

Based on coverage/deletion information (second method), conversion events were found in both directions across 882 Kbp of the D-genome reference sequence (Figure 1). These events ranged from 1 to 5470 bp, with a median of 337 bp (Figure S5). Two hundred fifty-nine regions suggested an A_T_-biased conversion. These regions spanned 275 Kbp and overlapped the coding sequence of 19 genes. They also included 12,696 putative homoeo-SNPs (based on the diploids), none of which was detected within the tetraploid. However, 1213 regions showed a D_T_-biased conversion. These regions spanned 607 Kbp and overlapped the coding sequence of 94 genes. They included 21,142 putative homoeo-SNPs, of which only three were also detected within the tetraploid. The genes overlapped by these regions had no enriched GO terms and indicated a conversion bias in both directions, but with the D_T_ direction most prominent, similar to that previously reported (Paterson *et al.* 2012). The events detected by the second method only included 1375 of the possibly conversion-related SNPs identified by the first method.

## Discussion

### Genome resources for *Gossypium*

Using the diploid re-sequencing data, we created several useful resources for the *Gossypium* genome. First, a genome-wide map of the SNPs between the diploid A-genomes and D-genomes of cotton was created. Re-sequencing multiple accessions of each diploid enabled us to distinguish bases that were specific to a single accession from bases that are more representative of one diploid genome or another. It also allowed us to identify conserved genomic features shared by all A-genome or D-genome species and accessions. In that sense, the multiple accessions of each species acted as re-sequencing replications of the A-genome or D-genome “treatments.” We have demonstrated that most SNPs identified between diploid genomes can be directly extrapolated to differences between the descendant allopolyploid genomes (*i.e.*, homoeo-SNPs) because of their recent common ancestor. In addition, we also identified several million homoeo-SNPs that were unique to the Maxxa allopolyploid genome. These documented SNPs can be used for genome identification of individual sequence reads (Udall 2006; Flagel *et al.* 2012) or the development of genotyping assays (Van Deynze *et al.* 2009; Byers *et al.* 2012). With an index of homoeo-SNPs, read-mapping efficiency was significantly improved and future false-positive allele-SNPs can be filtered out of marker sets, resulting in more reliable allele-SNP assessment. In addition to the homoeo-SNPs, we also identified allele-SNPs in the diploid cotton accessions. These SNP sets are available as Gbrowse tracks at CottonGen (http://www.cottongen.org) and as gff files at the Udall laboratory web site (http://udall-lab.byu.edu).

A second resource is the set of alignments of gene and protein sequences of the A-genomes, A_T_-genomes, and D_T_-genomes to accompany the previously published *G. raimondii* annotations (Paterson *et al.* 2012). These alignments were used to identify SNPs and to further refine our understanding of the molecular evolutionary differences between genomes. Because we have made this a public resource, any researcher investigating cotton now has homoeo-SNPs and allele-SNPs information for any target gene already identified. This simple, yet tedious, task has been a common obstacle of genetic research in polyploid cotton.

A third resource, and one that we suggest will be a fruitful topic for further investigation, is the description of putative duplications and deletions that distinguish the A-genomes and D-genomes and, hence, originated subsequent to their divergence from a common ancestor. These localized structural variations offer a rich source of sequences to mine for possible functional consequences, and to further our understanding of the mechanisms of copy number variation during genome evolution in plants.

Through our read-mapping efforts, we noticed that the limited number and the stochastic distribution of homoeo-SNPs could have implications for *de novo* genome assembly of polyploid cotton. Although 70% of the reads from allopolyploid cotton could be assigned to one of its two co-resident genomes, 30% of the reads that mapped to the D-genome reference did not overlap a homoeo-SNP and, hence, could not be categorized. Using an arbitrary length of 1000 bp, we found 47,399 unique loci where sequence reads of the A_T_-genome and D_T_-genome were indistinguishable when compared to each other and to the reference genome. Assuming sequence read lengths less than 500 bp, these regions would likely co-assemble during a *de novo* whole-genome shotgun assembly with current read lengths. Consequently, co-assembled segments will create unique challenges of graph structure bifurcation (or higher branching) during the contig construction steps of *de novo* assembly. Part of this challenge could be addressed by generating reads with a greater likelihood of overlapping homoeo-SNPs, *i.e.*, longer reads. Present data, however, suggest that *de novo* assembly of the allopolyploid cotton genome would not be successful if based on contemporary read lengths.

### Insights into the genome biology of *Gossypium*

One of the intriguing results of this study is the insight it provides into the origin and frequency of indels during A-genome and D-genome divergence. Because of the lack of an outgroup sequence, none of the “duplications” or “deletions” described here is polarized, so their duplicate or deleted status is only relative to the single D-genome reference. Moreover, the methods used do not yield insights into the mechanistic underpinnings of the indels, which may conceivably entail a full spectrum of deletional mechanisms and processes of tandem and dispersed duplication.

Notwithstanding, the present study does reveal the scope and scale of the indel generating process during 5–10 million years of diploid evolution. Additionally intriguing are the genomic distributions of the duplicated and deleted regions. For example, chromosome 13 is notable for its high frequency of duplications, containing one-sixth (2850/17,102) of the total number of conserved duplications in the A-genome, yet only 2.9% (174/6072) of the deletions. Given that chromosome 13 comprises a mere 7.8% of the *G. raimondii* reference sequence (Paterson *et al.* 2012), the suggestion arises that there has been exceptional expansion and/or contraction of this chromosome during the evolution of the two-fold size difference that distinguishes the A-genomes and D-genomes. Although some of this difference certainly reflects the expansion of transposable elements in the A-genome or possible contraction in the D-genome (Hawkins *et al.* 2009), it is unclear what genomic features have allowed more numerous rearrangements in chromosome 13 than within other chromosomes in the genome.

In addition to this broad-scale view of the contributions of duplications and deletions to *Gossypium* genome evolution, the data presented here offer a rich database that can be mined for potentially significant gene duplication and deletion. For example, gene loss has been associated with polyploidization (Shaked *et al.* 2001; Ozkan 2003; Han *et al.* 2005; Tate *et al.* 2009), but the deletions we have described in the A-genome occurred before polyploidization and include parts of approximately 1300 genes per accession. If these A-genome accessions were used as a "parental genome reference" for investigations of polyploidy, then the deletions common to the A_1_-genomes, A_2_-genomes, and ancestral A_T_-genomes would be confounded with any putative deletions that occurred as a result of polyploidization. Thus, this initial database of duplications and deletions will be a useful research tool for investigations of the evolution of the Gossypium genome.

We observed that several genes involved in starch synthesis were deleted in the A-genome diploids, including seven genes with 1,4-alpha-glucan branching enzyme activity (Table S3). It is tempting to speculate that these deletions increased the amount of glucose available in the A-genome diploid for cellulose synthesis and thereby played a role in the increased length of mature A-genome cotton fibers. Previous studies have documented altered carbon partitioning (Yong-Ling Ruan 1998) and altered starch accumulation (Chaohua *et al.* 2005) in fiberless *G. hirsutum* mutants. The deletion of starch genes in the A-genome may have been associated with the opposite effect, resulting in more carbon being allocated to cellulose production and less to starch production. We caution that many of the deleted genes are members of gene families and the remaining paralogs may partially or fully compensate for their deletion in the A-genome diploids. Nevertheless, the deleted genes discovered in this study offer interesting avenues of future research of gene duplication and functional compensation.

Among genes with altered stop codons, we detected an enriched number of genes having photosynthesis-related functions. It appears unlikely that the altered stop codons are attributable to horizontal transfer of chloroplast genes to the nucleus because only four have high similarity to chloroplast genes. Although a biological explanation for this enrichment remained a mystery, it was likely that a portion of the enrichment of photosynthesis GO terms was an artifact of the gene annotation process. For example, the actual stop codon location may have been ambiguous because the original annotation of these genes actually had no stop codon. Perhaps, the initial gene annotation effort was simply unable to identify the full coding sequence and subsequent updates will include corrections to the original annotations. Regardless of the initial annotation, the enrichment of photosynthesis GO terms in genes with altered stop codons was interesting, but it was not attributable to differences between the A-genomes, D-genomes, A_T_-genomes, and D_T_-genomes.

We also identified homogenized genome regions from conversion events between the two homoeologous genomes of *G. hirsutum*. Gene conversion has been defined as the nonreciprocal transfer of genetic information between homologous sequences, leading to homogenization during meiotic or mitotic recombination (Szostak *et al.* 1983; Chen *et al.* 2007; Hsu *et al.* 2010; Jacquemin *et al.* 2011). Unlike analyses of pairs of genes (Drouin 2002; Mondragon-Palomino and Gaut 2005; Xu *et al.* 2008), whole-genome analysis of gene conversion has been pioneered in rice (Xu *et al.* 2008; Wang *et al.* 2009; Jacquemin *et al.* 2011), a sequenced diploid genome with many closely related species also with sequenced genomes. By comparison of the diploid re-sequencing data to the publicly available WGS data of Maxxa Acala, we were able to identify conversion events between homoeologous sequences within the polyploid *Gossypium* genome using two different methods. The two methods of detecting homoeologous conversion events resulted in different directional biases.

Using the first method, we had previously used SNPs to estimate that up to 5% of the polyploid transcriptome had experienced “homoeologous gene conversion” (Salmon *et al.* 2010; Flagel *et al.* 2012). In both previous studies, identification of autapomorphic SNPs was not possible because of limited diploid sequencing data. Based on our current data, the presence of autapomorphic SNPs (and a liberal method of identification) appeared to have caused an overestimate in the amount of homoeologous conversion in genic regions. Thus, the genome sequence of a definitive outgroup is needed to unambiguously identify regions of conversion using SNP information alone. One dimension of the conversion events and the multi-alignment resource for all genes is the identification of loci where one of the two allopolyploid genomes has “overwritten” the other via a mechanism of reciprocal or nonreciprocal “gene conversion.” At present, the functional consequences of these observations remain unexplored, but it is intriguing to ask whether these conversion events are functionally insignificant (which might, for example, be the case when only synonymous sites are involved) or if, instead, specific genes or regulatory sequences have been selectively “doubled” or “eliminated” by this unusual intergenomic aspect of allopolyploid speciation.

This first method contains an inherent bias in favor of A_T_-biased conversion because of the greater genetic distance between D_T_ and D_5_ compared to the distance between A_T_ and A_1_ or A_2_. This bias, however, should only be approximately 50% based on our understanding of the genetic distances between A and A_T_ *vs.* D and D_T_ (the latter is 50% greater) (Flagel *et al.* 2012). In addition, the genotype pattern indicative of a conversion is indistinguishable from that caused by an autapomorphic SNP in the diploid species. For example, suppose an autapomorphic SNP in the A-genome ancestor of A_1_ and A_2_ (not shared with A_T_) changes a C to a T at a given locus. Consequently, the D-genome diploids, D_T_-genome, and A_T_-genome would all have a C, whereas the A-genome diploids would have a T. So, the A_T_ nucleotide would appear the same as D and D_T_, suggesting a D_T_-biased allele conversion event, even though they simply shared the ancestral allele. These confounding autapomorphic SNPs would have occurred after the divergence of A_T_ but before the A_1_–A_2_ split, suggesting a D_T_-biased conversion. However, an A_T_-biased conversion would be suggested by an autapomorphic SNP occurring after the divergence of D_T_, but before the most recent common ancestor of the D_5_ diploids. Because the A-genome diploid is approximately a two-fold better approximation of the actual progenitor diploid than is the D-genome diploid (Wendel *et al.* 2012), these branch lengths are different (Figure 3). In fact, the phylogeny showed a distance of only 0.00447 for the branch corresponding to an autapomorphic SNP shared by all A diploids but not by A_T_. However, the equivalent branch in the D-genome clade has an autapomorphic SNP distance of 0.05385. These numbers suggest that, in the absence of any actual conversion events, there should be more than 12-times (0.05385/0.00447) as many SNPs that look like A_T_-biased conversion—because they are shared by A diploids but not by A_T_—as SNPs that look like D_T_-biased conversion. The difference between the 12-fold expected value for A_T_-biased conversions as visualized by the branch lengths to the five-fold observed value could be explained by a bias toward D_T_-biased conversion events, as reported elsewhere and as detected by the second coverage-based method. Thus, we consider the conversion events detected by the SNP-based method (method 1) to be inaccurate based on autapomorphic SNPs, but we consider the conversion events of the coverage method (method 2) to be a conservative, yet relatively accurate, assessment of conversion between the polyploid *G. hirsutum* genomes.

The second method of conversion detection used deletion and coverage information to detect many separate events, and the direction of bias agreed with previous reports (Paterson *et al.* 2012). This method was very conservative and may represent a minimum amount of conversion events in the polyploid genome because of the uncertainty of the actual endpoints of conversion and the additional amounts of conversion suggested between homoeologous gene copies in the dN/dS analysis. The conversion events resulted in a loss of genomic diversity between the A_T_- and D_T_-genomes. Parts of at least 113 genes were included in conversion events between homoeologous chromosomes. Other investigations of genome evolution in rice have uncovered convergent evolution of ancient paralogs on Chr11 and Chr12 (∼2.1 Mb) mediated by gene conversion including up to 180 genes (Jacquemin *et al.* 2011). The conversion events we have described were more recent (after polyploidization 1–2 Ma) and our inference space was limited to a single species of *G. hirsutum*. It will be interesting to see if other polyploid Gossypium genomes also have the same conversion events in their genomes, and to estimate the rate of gene conversion between homoeologous genomes.

Whole-genome re-sequencing of diploid *Gossypium* has identified insights into the genome evolution of cotton. These insights proved to be useful for characterization of the *G. hirsutum* genome via publicly available re-sequencing data. Additional *de novo* and re-sequencing efforts of polyploid Gossypium will continue to add to our understanding of the cotton genome, thereby enhancing our ability to manipulate the fiber and agronomic characteristics of cotton.

## Supplementary Material

Supporting Information
