# Supplementary material for: Insights into the Evolution of Cotton Diploids and Polyploids from Whole-Genome Re-sequencing
Source: G3 (Bethesda). 2013 Oct 1;3(10):1809–18. doi: 10.1534/g3.113.007229 (PMC3789805; doi:10.1534/g3.113.007229)
Supplement: Supporting Information [file supp_3_10_1809__index.html]

Insights into the Evolution of Cotton Diploids and Polyploids from Whole-Genome Re-sequencing — Supporting Information 

# Insights into the Evolution of Cotton Diploids and Polyploids from Whole-Genome Re-sequencing

## Supporting Information for Page *et al.*, 2013

**Files in this Data Supplement:**

- Supporting Information - Figures S1-S5 and Tables S1-S3 (PDF, 1 MB)
- Figure S1 - PolyCat categorization of reads (PDF, 434 KB)
- Figure S2 - Histogram of the number of homoeo-SNPs (x-axis) per gene (y-axis). (PDF, 1 MB)
- Figure S3 - The length distribution of genes without any SNPs and present in both genomes (red; N = 378) and the length distribution of all annotated genes (blue; N = 37,223). (PDF, 496 KB)
- Figure S4 - A summary of the genomic deletions detected in the A-genome when sequencing reads were mapped to the D-genome reference. (PDF, 419 KB)
- Figure S5 - A histogram of estimated lengths of regions of genome conversion (PDF, 484 KB)
- Table S1 - Lists of GO terms enriched in genes without stop codons: D5 reference (a), pseudo-A (b), pseudo-AT (c), pseudo-DT (d). (PDF, 453 KB)
- Table S2 - Number of SNPs between each pair of accessions (PDF, 410 KB)
- Table S3 - Table of genes (a) and GO terms (b) enriched in A-genome deleted genes. (.xlsx, 115 KB)
