# Supplementary material for: Insights into the Evolution of Cotton Diploids and Polyploids from Whole-Genome Re-sequencing
Source: G3 (Bethesda). 2013 Oct 1;3(10):1809–18. doi: 10.1534/g3.113.007229 (PMC3789805; doi:10.1534/g3.113.007229)
Supplement: Supporting Information [file supp_g3.113.007229_FigureS1.pdf]

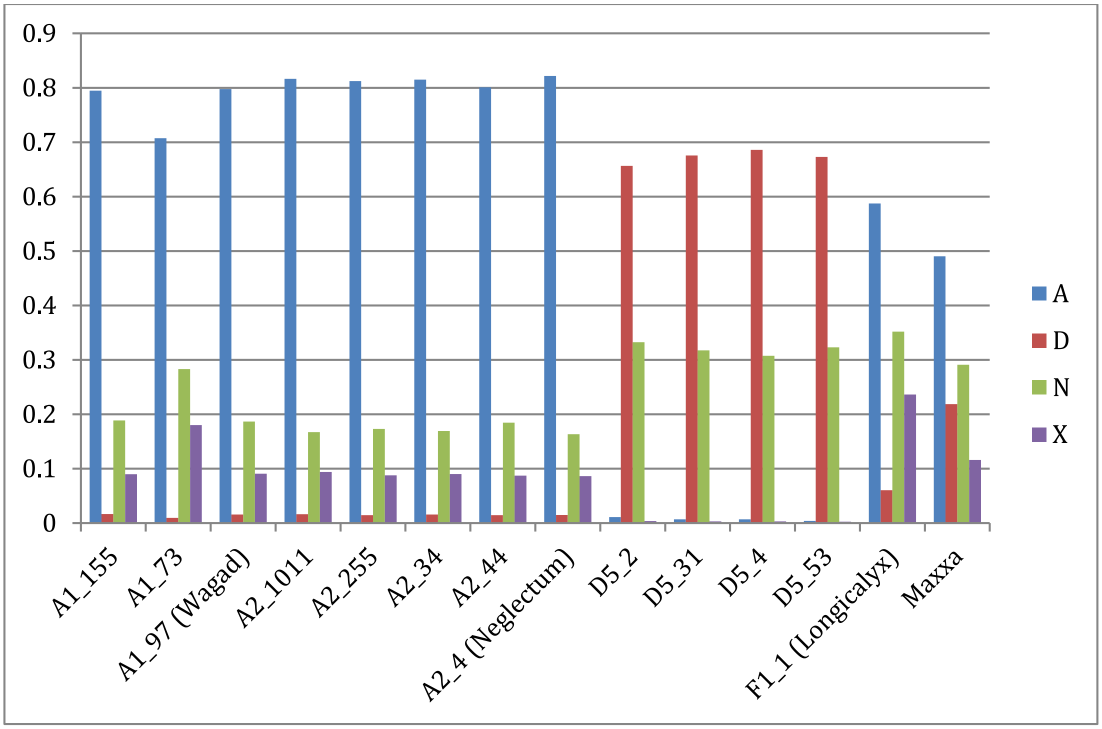

Figure S1 PolyCat categorization of reads. For each accession, the fraction of reads categorized as A-genome (blue), D-genome (red), uncategorizable (green), and chimeric (purple). The uncategorizable reads are a superset of the chimeric reads.
