# Supplementary figures and images for: Insights into the Evolution of Cotton Diploids and Polyploids from Whole-Genome Re-sequencing
Source: G3 (Bethesda). 2013 Oct 1;3(10):1809–18. doi: 10.1534/g3.113.007229 (PMC3789805; doi:10.1534/g3.113.007229)

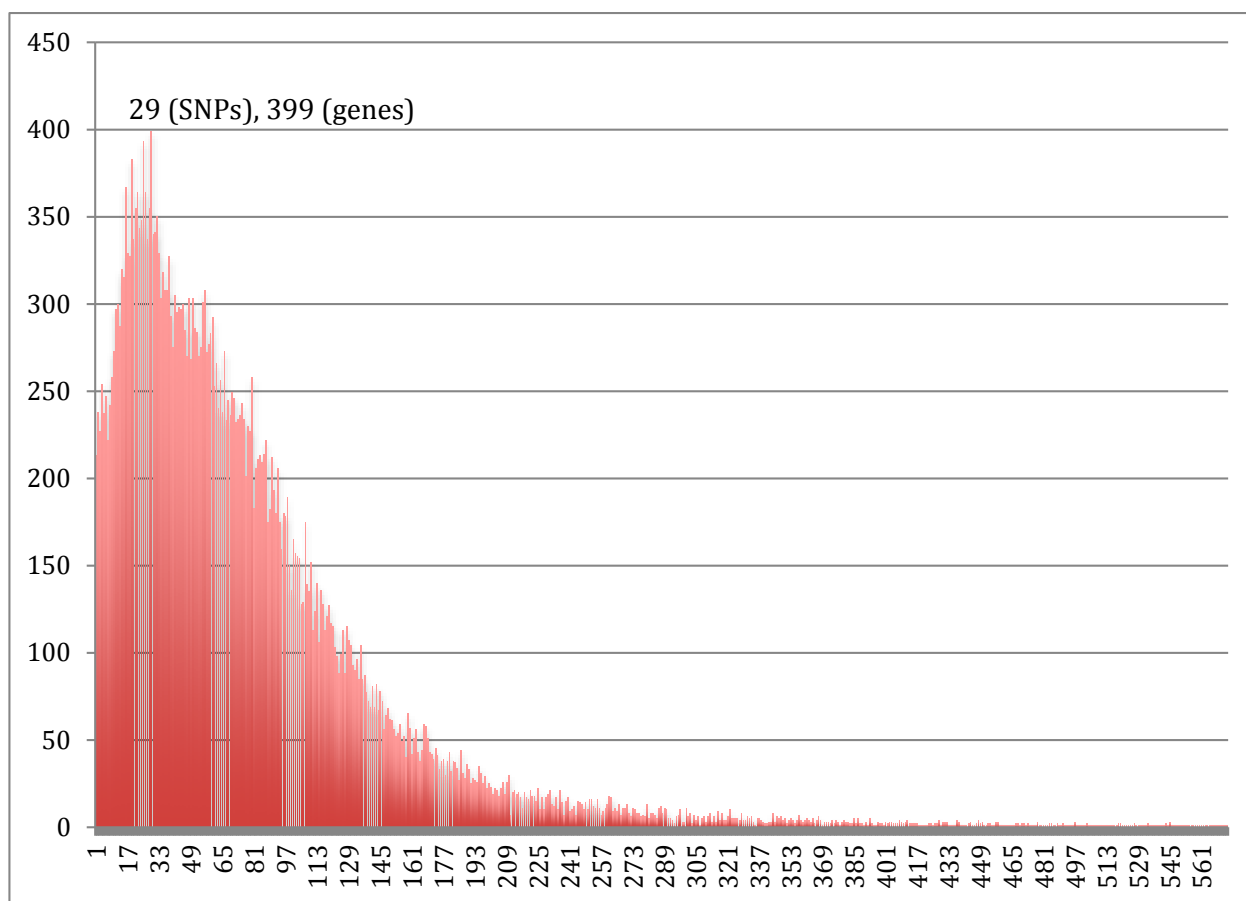

**Figure S2** Histogram of the number of homoeo-SNPs (x-axis) per gene (y-axis). Most genes had 30-40 homoeo-SNPs.

Supplement: Supporting Information [file supp_g3.113.007229_FigureS2.pdf]

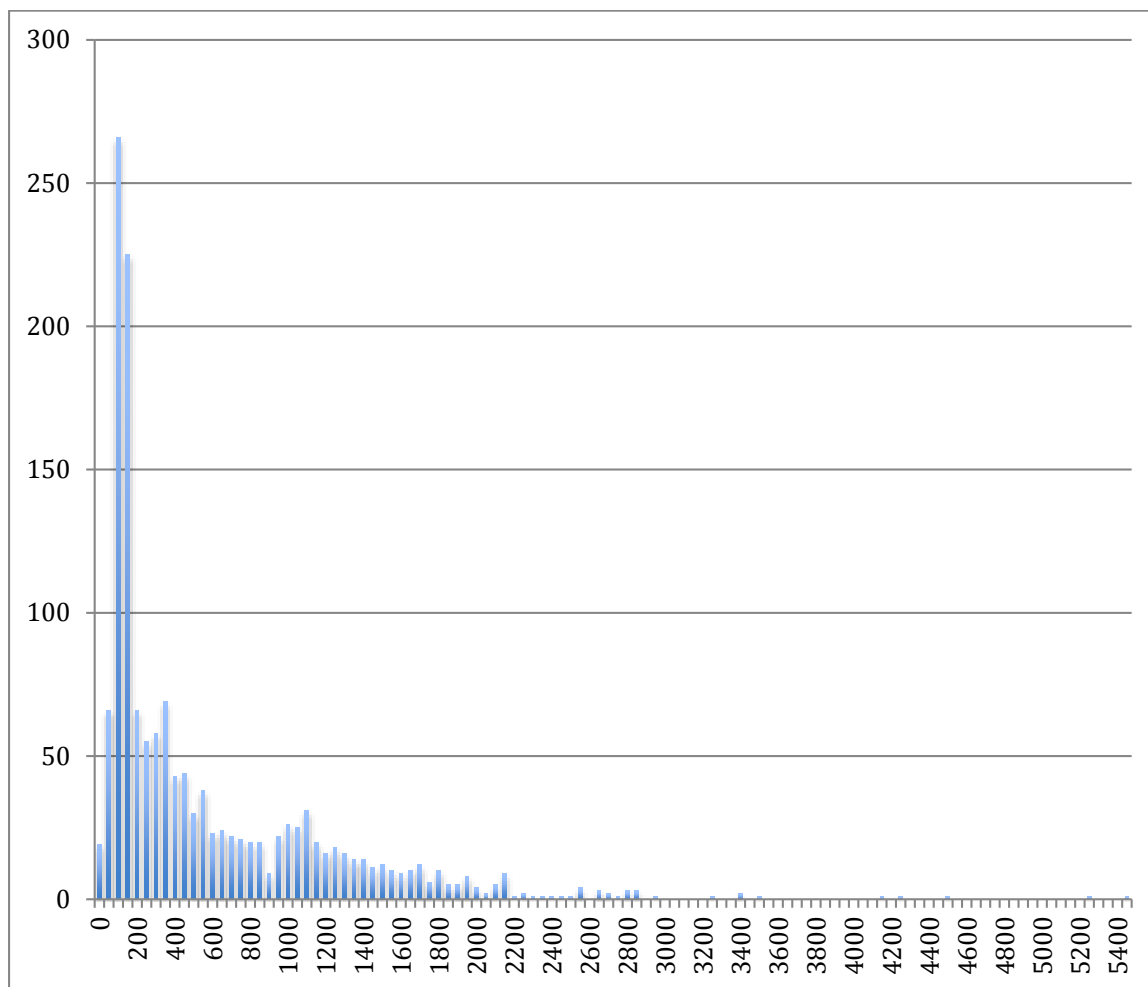

**Figure S5** A histogram of estimated lengths of regions of genome conversion.

Supplement: Supporting Information [file supp_g3.113.007229_FigureS5.pdf]
