# Supplementary material for: Insights into the Evolution of Cotton Diploids and Polyploids from Whole-Genome Re-sequencing
Source: G3 (Bethesda). 2013 Oct 1;3(10):1809–18. doi: 10.1534/g3.113.007229 (PMC3789805; doi:10.1534/g3.113.007229)
Supplement: Supporting Information [file supp_g3.113.007229_FigureS3.pdf]

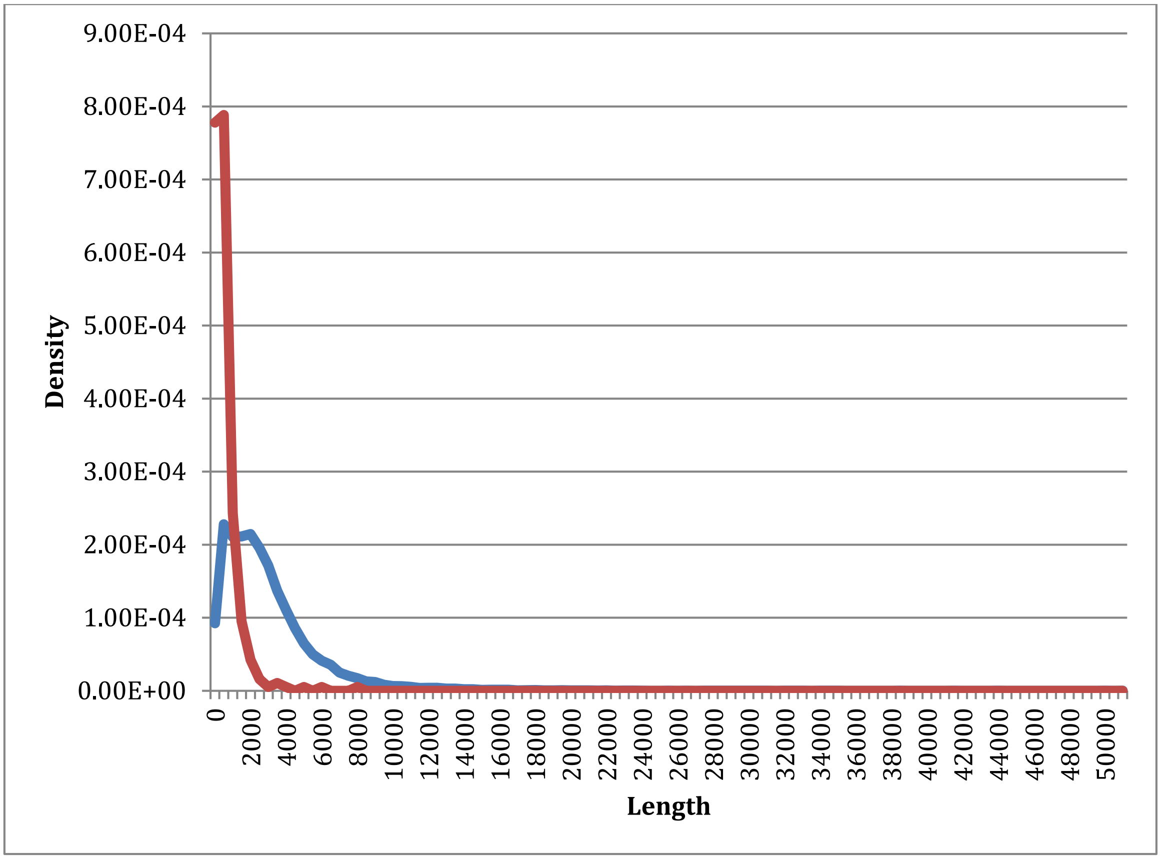

Figure S3 The length distribution of genes without any SNPs and present in both genomes (red; N = 378) and the length distribution of all annotated genes (blue; N = 37,223).
