# Supplementary material for: Insights into the Evolution of Cotton Diploids and Polyploids from Whole-Genome Re-sequencing
Source: G3 (Bethesda). 2013 Oct 1;3(10):1809–18. doi: 10.1534/g3.113.007229 (PMC3789805; doi:10.1534/g3.113.007229)
Supplement: Supporting Information [file supp_g3.113.007229_FigureS4.pdf]

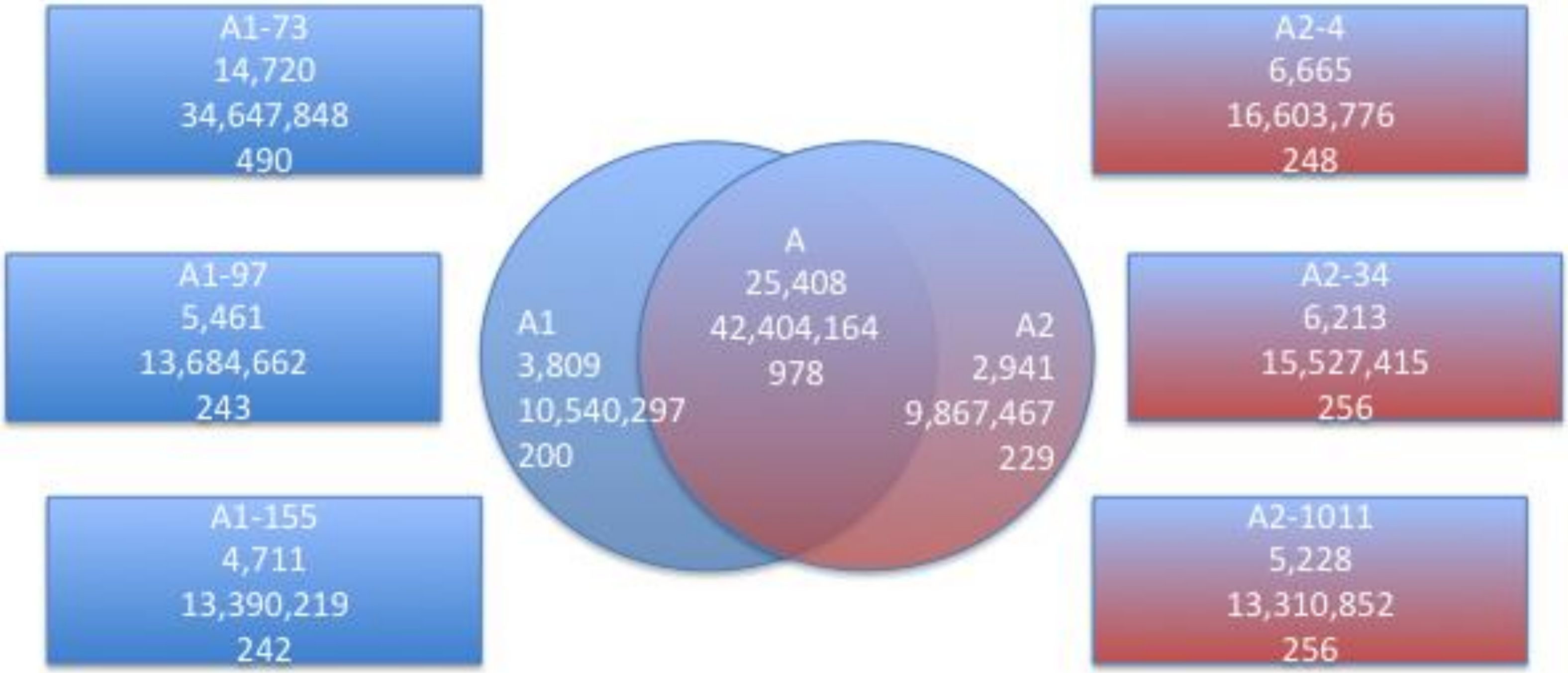

.

Figure S4 A summary of the genomic deletions detected in the A-genome when sequencing reads were mapped to the D-genome reference. Each shape contains 1) accession ID, 2) the total number of deletions, 3) the sum total length of deletions in base-pairs, and 4) the number of deleted genes. Blue shapes indicate A_1, and blue shapes indicate A_2.
