# Supplementary material for: Insights into the Evolution of Cotton Diploids and Polyploids from Whole-Genome Re-sequencing
Source: G3 (Bethesda). 2013 Oct 1;3(10):1809–18. doi: 10.1534/g3.113.007229 (PMC3789805; doi:10.1534/g3.113.007229)
Supplement: Supporting Information [file supp_g3.113.007229_TableS1.pdf]

Table S1 Lists of GO terms enriched in genes without stop codons: D_5 reference (a), pseudo-A (b), pseudo-A_T (c), pseudo-D_T (d).

a)

GO Term	Name	Type	FDR	single test p-Value	# in test group	# in reference group	# non annot test	# non annot reference group	Over/Under
GO:0015979	photosynthesis	P	6.8E-10	9.3E-14	23	324	209	25464	over
GO:0022900	electron transport chain	P	0.000000046	1.3E-11	20	301	212	25487	over
GO:0034357	photosynthetic membrane	C	0.00000067	2.7E-10	26	634	206	25154	over
GO:0019684	photosynthesis, light reaction	P	0.0000012	8.2E-10	15	196	217	25592	over
GO:0044436	thylakoid part	C	0.0000012	8.5E-10	26	670	206	25118	over
GO:0009767	photosynthetic electron transport chain	P	0.0000029	2.4E-09	11	93	221	25695	over
GO:0006091	generation of precursor metabolites and energy	P	0.0000037	3.5E-09	24	615	208	25173	over
GO:0042651	thylakoid membrane	C	0.000015	0.000000016	23	615	209	25173	over
GO:0009521	photosystem	C	0.000021	0.000000026	11	119	221	25669	over
GO:0018298	protein-chromophore linkage	P	0.000086	0.00000012	6	21	226	25767	over
GO:0009535	chloroplast thylakoid membrane	C	0.000086	0.00000014	21	585	211	25203	over
GO:0055035	plastid thylakoid membrane	C	0.000086	0.00000014	21	586	211	25202	over
GO:0009579	thylakoid	C	0.00017	0.0000003	26	906	206	24882	over
GO:0016168	chlorophyll binding	F	0.00039	0.00000074	7	50	225	25738	over
GO:0016772	transferase activity, transferring phosphorus-containing groups	F	0.002	0.0000042	49	2788	183	23000	over
GO:0009534	chloroplast thylakoid	C	0.0023	0.0000053	21	741	211	25047	over
GO:0031976	plastid thylakoid	C	0.0023	0.0000053	21	741	211	25047	over
GO:0009523	photosystem II	C	0.0025	0.0000063	7	71	225	25717	over
GO:0031984	organelle subcompartment	C	0.0025	0.0000065	21	751	211	25037	over
GO:0009769	photosynthesis, light harvesting in photosystem II	P	0.005	0.000014	3	3	229	25785	over
GO:0009507	chloroplast	C	0.0079	0.000023	53	3316	179	22472	over
GO:0032991	macromolecular complex	C	0.021	0.000064	47	2923	185	22865	over
GO:0003824	catalytic activity	F	0.021	0.000065	145	12823	87	12965	over
GO:0009055	electron carrier activity	F	0.05	0.00016	14	483	218	25305	over

b)

GO Term	Name	Type	FDR	single test p-Value	# in test group	# in reference group	# non annot test	# non annot reference group	Over/Under
GO:0015979	photosynthesis	P	0.00000025	3.4E-11	28	319	434	25239	over
GO:0022900	electron transport chain	P	0.00000058	1.6E-10	26	295	436	25263	over
GO:0009767	photosynthetic electron transport chain	P	0.000011	4.7E-09	14	90	448	25468	over
GO:0019684	photosynthesis, light reaction	P	0.000016	8.5E-09	19	192	443	25366	over
GO:0006091	generation of precursor metabolites and energy	P	0.00078	0.00000053	31	608	431	24950	over
GO:0034357	photosynthetic membrane	C	0.0013	0.0000011	31	629	431	24929	over
GO:0044436	thylakoid part	C	0.0033	0.0000031	31	665	431	24893	over
GO:0009521	photosystem	C	0.0034	0.0000037	12	118	450	25440	over
GO:0018298	protein-chromophore linkage	P	0.0052	0.0000065	6	21	456	25537	over
GO:0016168	chlorophyll binding	F	0.0052	0.0000072	8	49	454	25509	over
GO:0016772	transferase activity, transferring phosphorus-containing groups	F	0.0073	0.000011	81	2756	381	22802	over
GO:0042651	thylakoid membrane	C	0.0075	0.000012	28	610	434	24948	over
GO:0009535	chloroplast thylakoid membrane	C	0.02	0.000037	26	580	436	24978	over
GO:0055035	plastid thylakoid membrane	C	0.02	0.000038	26	581	436	24977	over
GO:0003824	catalytic activity	F	0.025	0.000052	272	12696	190	12862	over
GO:0009579	thylakoid	C	0.031	0.000067	34	898	428	24660	over
GO:0009523	photosystem II	C	0.032	0.000074	8	70	454	25488	over
GO:0009769	photosynthesis, light harvesting in photosystem II	P	0.043	0.00011	3	3	459	25555	over

c)

GO Term	Name	Type	FDR	single test p-Value	# in test group	# in reference group	# non annot test	# non annot reference group	Over/Under
GO:0015979	photosynthesis	P	3.6E-09	4.9E-13	28	319	359	25314	over
GO:0022900	electron transport chain	P	0.000000071	1.9E-11	25	296	362	25337	over
GO:0019684	photosynthesis, light reaction	P	0.00000089	4.7E-10	19	192	368	25441	over
GO:0009767	photosynthetic electron transport chain	P	0.00000089	4.9E-10	14	90	373	25543	over
GO:0034357	photosynthetic membrane	C	0.00011	0.000000073	30	630	357	25003	over
GO:0006091	generation of precursor metabolites and energy	P	0.00015	0.00000013	29	610	358	25023	over
GO:0044436	thylakoid part	C	0.00024	0.00000023	30	666	357	24967	over
GO:0009521	photosystem	C	0.00054	0.00000059	12	118	375	25515	over
GO:0042651	thylakoid membrane	C	0.0011	0.0000013	27	611	360	25022	over
GO:0016168	chlorophyll binding	F	0.0014	0.0000019	8	49	379	25584	over
GO:0018298	protein-chromophore linkage	P	0.0016	0.0000024	6	21	381	25612	over
GO:0009579	thylakoid	C	0.0027	0.0000044	33	899	354	24734	over
GO:0009535	chloroplast thylakoid membrane	C	0.0028	0.0000051	25	581	362	25052	over
GO:0055035	plastid thylakoid membrane	C	0.0028	0.0000053	25	582	362	25051	over
GO:0009523	photosystem II	C	0.01	0.000021	8	70	379	25563	over
GO:0009769	photosynthesis, light harvesting in photosystem II	P	0.029	0.000063	3	3	384	25630	over
GO:0009534	chloroplast thylakoid	C	0.034	0.000085	26	736	361	24897	over
GO:0031976	plastid thylakoid	C	0.034	0.000085	26	736	361	24897	over
GO:0031984	organelle subcompartment	C	0.04	0.0001	26	746	361	24887	over
GO:0055114	oxidation-reduction process	P	0.045	0.00012	57	2268	330	23365	over

d)

GO Term	Name	Type	FDR	single test p-Value	# in test group	# in reference group	# non annot test	# non annot reference group	Over/Under
GO:0015979	photosynthesis	P	4.3E-10	5.9E-14	24	323	227	25446	over
GO:0022900	electron transport chain	P	0.00000019	5.3E-11	20	301	231	25468	over
GO:0034357	photosynthetic membrane	C	0.00000077	3.1E-10	27	633	224	25136	over
GO:0044436	thylakoid part	C	0.0000018	0.000000001	27	669	224	25100	over
GO:0019684	photosynthesis, light reaction	P	0.0000035	2.4E-09	15	196	236	25573	over
GO:0009521	photosystem	C	0.0000057	5.3E-09	12	118	239	25651	over
GO:0009767	photosynthetic electron transport chain	P	0.0000057	5.5E-09	11	93	240	25676	over
GO:0042651	thylakoid membrane	C	0.000014	0.000000016	24	614	227	25155	over
GO:0006091	generation of precursor metabolites and energy	P	0.000014	0.000000017	24	615	227	25154	over
GO:0009535	chloroplast thylakoid membrane	C	0.000083	0.00000012	22	584	229	25185	over
GO:0055035	plastid thylakoid membrane	C	0.000083	0.00000013	22	585	229	25184	over
GO:0018298	protein-chromophore linkage	P	0.00012	0.00000019	6	21	245	25748	over
GO:0009579	thylakoid	C	0.00023	0.00000041	27	905	224	24864	over
GO:0016168	chlorophyll binding	F	0.00065	0.0000013	7	50	244	25719	over
GO:0009534	chloroplast thylakoid	C	0.0025	0.0000054	22	740	229	25029	over
GO:0031976	plastid thylakoid	C	0.0025	0.0000054	22	740	229	25029	over
GO:0031984	organelle subcompartment	C	0.0028	0.0000066	22	750	229	25019	over
GO:0009523	photosystem II	C	0.0043	0.000011	7	71	244	25698	over
GO:0009769	photosynthesis, light harvesting in photosystem II	P	0.0066	0.000017	3	3	248	25766	over
GO:0016772	transferase activity, transferring phosphorus-containing groups	F	0.0066	0.000018	50	2787	201	22982	over
GO:0003824	catalytic activity	F	0.011	0.000031	157	12811	94	12958	over
GO:0047746	chlorophyllase activity	F	0.031	0.000093	2	0	249	25769	over
