# Supplementary material for: Insights into the Evolution of Cotton Diploids and Polyploids from Whole-Genome Re-sequencing
Source: G3 (Bethesda). 2013 Oct 1;3(10):1809–18. doi: 10.1534/g3.113.007229 (PMC3789805; doi:10.1534/g3.113.007229)
Supplement: Supporting Information [file supp_g3.113.007229_TableS2.pdf]

Table S2 Number of SNPs between each pair of accessions. See Figure 3.

	F1_1	A1_73	A1_97	A1_155	A2_4	A2_34	A2_255	A2_1011	Maxxa.A	Maxxa.D	D5_2	D5_4	D5_31	D5_53
F1_1	-	17,564,992	21,305,598	22,079,020	21,859,715	21,674,508	20,679,406	21,408,246	20,291,884	29,073,437	33,767,585	34,170,146	34,562,572	34,557,441
A1_73	-	-	3,631,045	3,900,567	6,303,268	6,215,005	6,012,594	6,207,091	6,300,220	22,400,730	24,134,306	24,443,485	24,723,105	24,722,179
A1_97	-	-	-	4,946,396	7,328,205	7,031,582	7,073,657	7,155,650	7,780,968	27,700,097	30,348,697	30,730,738	31,096,279	31,093,916
A1_155	-	-	-	-	7,403,173	7,229,834	7,156,740	7,560,136	7,998,112	28,828,413	31,885,907	32,291,549	32,693,266	32,691,124
A2_4	-	-	-	-	-	3,716,422	3,707,066	4,168,039	7,903,834	28,578,818	31,594,908	31,999,160	32,392,725	32,391,137
A2_34	-	-	-	-	-	-	3,728,031	3,850,083	7,864,907	28,272,425	31,234,868	31,581,814	31,889,687	31,886,283
A2_255	-	-	-	-	-	-	-	4,013,599	7,354,551	27,224,622	30,223,121	30,521,981	30,788,394	30,784,707
A2_1011	-	-	-	-	-	-	-	-	7,887,558	27,866,733	30,820,039	31,120,155	31,340,231	31,338,170
Maxxa.A	-	-	-	-	-	-	-	-	-	26,728,016	29,477,752	29,839,383	30,196,553	30,191,703
Maxxa.D	-	-	-	-	-	-	-	-	-	-	8,277,247	8,374,774	8,468,863	8,465,441
D5_2	-	-	-	-	-	-	-	-	-	-	-	224,283	218,142	223,665
D5_4	-	-	-	-	-	-	-	-	-	-	-	-	235,864	244,890
D5_31	-	-	-	-	-	-	-	-	-	-	-	-	-	78,686
D5_53	-	-	-	-	-	-	-	-	-	-	-	-	-	-
